# PredictION: a predictive model to establish the performance of Oxford sequencing reads of SARS-CoV-2

**DOI:** 10.7717/peerj.14425

**Published:** 2022-11-30

**Authors:** David E. Valencia-Valencia, Diana Lopez-Alvarez, Nelson Rivera-Franco, Andres Castillo, Johan S. Piña, Carlos A. Pardo, Beatriz Parra

**Affiliations:** 1 Laboratorio de Técnicas y Análisis Ómicos—TAOLab/CiBioFi, Facultad de Ciencias Naturales y Exactas, Universidad del Valle, Cali, Valle del Cauca, Colombia; 2 Departamento de Ciencias Biológicas, Facultad de Ciencias Agropecuarias, Universidad Nacional de Colombia, Palmira, Valle del Cauca, Colombia; 3 Grupo VIREM—Virus Emergentes y Enfermedad, Escuela de Ciencias Básicas, Facultad de Salud, Universidad del Valle, Cali, Valle del Cauca, Colombia; 4 Department of Data Science, People Contact, Manizales, Caldas, Colombia; 5 Department of Neurology, Pathology, Johns Hopkins University School of Medicine, Baltimore, MD, United States of America

**Keywords:** Oxford nanopore technologies, Genomes, Machine learning, Sequences, Linear models

## Abstract

The optimization of resources for research in developing countries forces us to consider strategies in the wet lab that allow the reuse of molecular biology reagents to reduce costs. In this study, we used linear regression as a method for predictive modeling of coverage depth given the number of MinION reads sequenced to define the optimum number of reads necessary to obtain >200X coverage depth with a good lineage-clade assignment of SARS-CoV-2 genomes. The research aimed to create and implement a model based on machine learning algorithms to predict different variables (*e.g.*, coverage depth) given the number of MinION reads produced by Nanopore sequencing to maximize the yield of high-quality SARS-CoV-2 genomes, determine the best sequencing runtime, and to be able to reuse the flow cell with the remaining nanopores available for sequencing in a new run. The best accuracy was −0.98 according to the R squared performance metric of the models. A demo version is available at https://genomicdashboard.herokuapp.com/.

## Introduction

The Oxford Nanopore Technologies (ONT) MinION sequencing platform provides a method for high-throughput and cost-effective long-read sequencing in a portable device (Gauthier et al., 2021), and has become a fast and reliable tool for epidemiological surveillance of severe acute respiratory syndrome coronavirus 2 (SARS-CoV-2). During the COVID-19 pandemic ONT accelerated the production of genome data worldwide (As of October 15, 2022 there are about 13.8 million SARS-CoV-2 genomes submitted to the Global Initiative on Sharing All Influenza Data (GISAID) database), allowing for characterization of different lineages or variants and providing an essential tool for effective health policy decision making. The most widely adopted targeted amplicon approach for SARS-CoV-2 genomic sequencing is the ARTIC protocol (Lambisia et al., 2022).

Despite its multiple advantages for clinical and epidemiological applications, this sequencing technology is very expensive, especially for the public health systems in developing countries. The estimated costs of reagents and consumables range between $11.50 to $35.88 for one sample when calculated based on 96 samples per sequencing run (Lambisia et al., 2022) plus $900 per flow cell. Therefore, optimized one-time reuse of the MinION flow cells is a feasible cost-effective alternative that provides an opportunity to perform another additional experiment using the same set of barcodes with a different type of sequencing approach/targets. The idea would be to stop sequencing once enough reads are attained for optimal genome assembly, leaving a remaining number of nanopores still available for sequencing in a new experiment. In fact, Oxford Nanopore provides flow cell wash buffers and storage buffers that facilitate the storage of second-use flow cells. The implementation of machine learning algorithms can help optimize different variables involved in obtaining a good quality SARS-CoV-2 genome from a certain number of sequenced reads. Linear Regression is a statistical model well-known in Supervised Machine Learning (SML), and it is applied to establish the relationship between a dependent variable and one or more independent variables since the algorithm is trained on both input features and output labels (Osisanwo et al., 2017).

In addition to generating value and exploiting data potential, data science applications should allow visualization and manipulation of the results obtained from the analysis (Verbert et al., 2014). Therefore, we also propose an online monitoring model that provides other researchers with a helping hand for their experiments, since the Web applications enhance the software performance, availability, and scalability (Verbert et al., 2013). In this study, different machine learning algorithms were implemented to make predictions of the optimal number of sequenced reads needed to obtain good coverage depth (>200X) using the MinION sequencing platform and lab-scale data for SARS-CoV-2 genomes. Additionally, we designed a Machine Learning application by loading the best model into dynamic dashboards that allow users to modify and interact with the model’s inputs, apply filters, and visualize graphics interactively.

## Materials & Methods

### Data collection and processing

We used five variables: number of sequenced reads per genome, CT (Cycle threshold) value (N2 target gene; N is the Nucleocapside protein gene and number 2 is a second specific sequence targeted within the N gene), mean coverage depth, coverage genome (percentage), and quantification cDNA (ng/µl) for a dataset (1) that included 1461 samples without CT values, and a dataset (2) with 471 samples that was a subset of dataset 1 (Table S1) that included CT values. Data were generated in amplicon-targeted sequencing experiments for SARS-CoV-2 genomes assemblies using ARTIC Network’s protocol V3 (Tyson et al., 2020) and a MinION device. This protocol of primer sets, and amplicons is one of the most widely used SARS-CoV-2 sequencing protocols (Tyson et al., 2020). To understand the meaning and the predictive power of the variables we conducted exploratory analysis before modeling, as follows: (i) The number of reads data were grouped into bins and the means (and medians) of coverage depth in each bin were compared; if coverage depth was similar across bins, the number of reads would be non-predictive. (ii) We plotted the number of reads against coverage depth. These plots were generated using Python (v3.9.9) and libraries Pandas (v1.4.4), NumPy (v1.23.3), Matplotlib (v3.5.3), and Seaborn (v0.12.0).

### Model building

The primary independent variable was “sequenced reads”, while the remaining variables (*e.g.*, CT, cDNA) were used to describe and proceed with the design, training, testing, and evaluation of the SML model. Both datasets were scaled using the RobustScaler function from Sklearn (v1.1.1), then the datasets were split into train (75%) and test (25%) sets. For reproducibility purposes, a random seed of 27 was set for all models that used a random state.

All models were trained with both datasets to select the best performance estimator. We use Lasso (LssR), Gradient Boosting Regressor (GBR), Random Forest Regressor (RFR), and Support Vector Regressor (SVR) as SML algorithms to predict continuous-valued outputs. LssR is a linear model regression that estimates sparse coefficients based on L1 penalization (Cherkassky & Ma, 2003). On the other hand, both RFR and GBR are ensemble methods that combine multiple learning algorithms to get a better performance prediction. Specifically, GBR builds an additive model in a forward stage-wise fashion and each stage fits a regression tree on the negative gradient of the given loss function. RFR fits a set number of decision trees of subsamples of a dataset to improve the predictive power. Lastly, SVR is an SML algorithm that analyses data regression based on a hyperplane and support vectors (Smola & Schölkopf, 2004); this method considers the points that are within the decision boundary line and fit the error within a certain threshold. The best fit line is the hyperplane that optimizes classification. Model hyperparameters were modified to achieve better performance for models. LssR was trained with different values for *alpha* regularization; those values were 200 numbers on a log scale from −10 to 3. For RFR and GBR, random seeds were set at 27. For SVR default hyperparameters were used. All algorithms were implemented using Python v3.9.9 and Sklearn (v1.1.1). Additionally, to compare the models, we used R squared as our metric for model performance with k-fold cross-validation splitting the dataset into five random folds to avoid model overfitting. Additionally, we reported the average of the five scores provided by the cross-validation process.

Taking into account that all variables are continuous, a Pearson correlation coefficient was calculated as a measure of the strength of the relationship between them; this kind of analysis was carried out on both datasets to decide which features to keep or discard according to predictive power. To aid the reproducibility of this work, the code was uploaded to an open access repository on GitHub (https://github.com/TAOLabUV/PredictION) as well as selected datasets.

### Performance metrics

**Figure 1:**
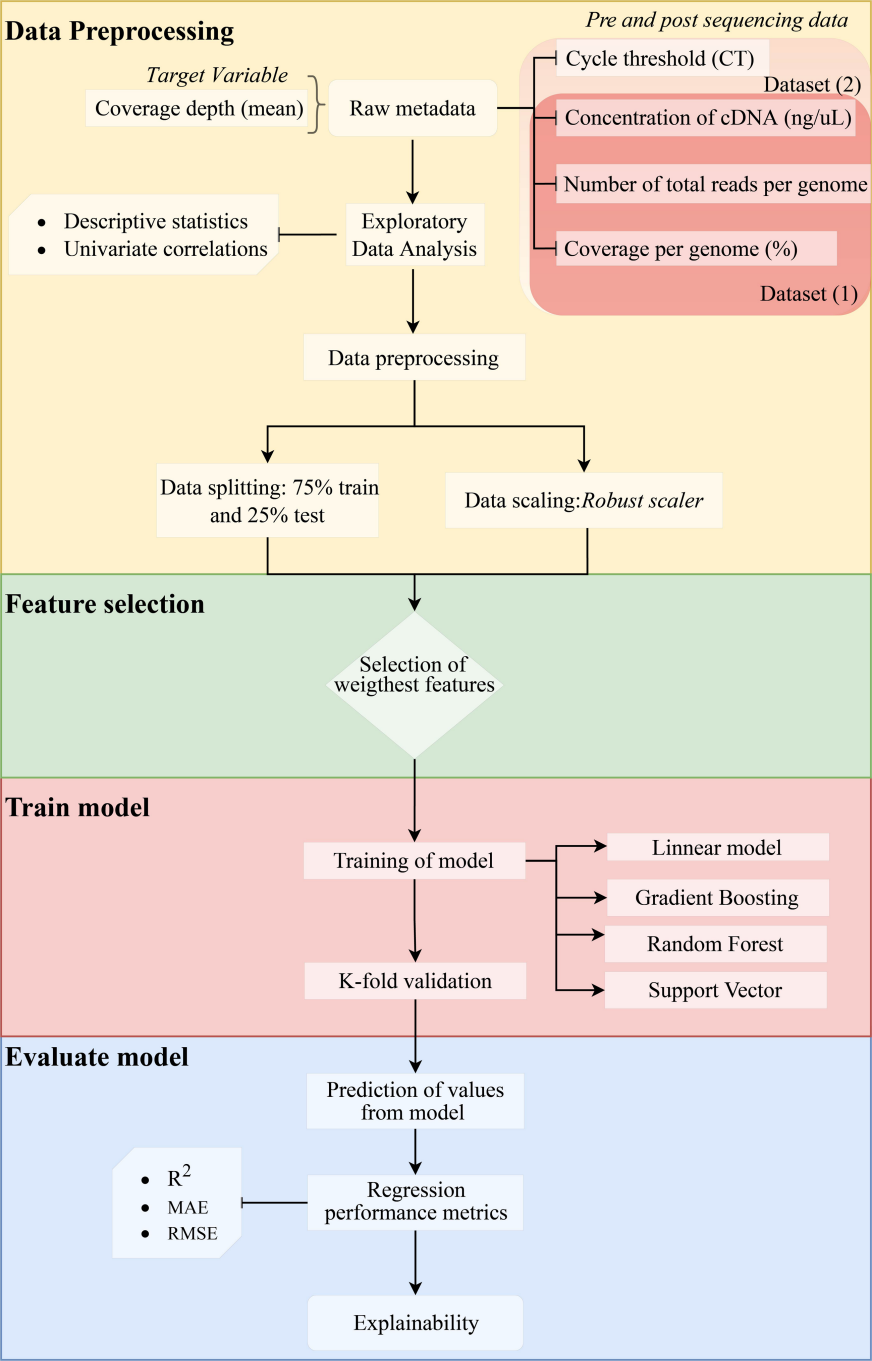
Methodology flowchart for an obtained optimal model.

We assessed the efficiency of the model using the coefficient of determination (R^2^) (1), mean absolute error (MAE) (2), and root mean squared error (RMSE) (3). Since errors can be both positive (actual > prediction) and negative (actual < prediction), we measure the absolute value and the squared value of each error. The R^2^, MAE, and RMSE are computed as follows:

(1) \begin{eqnarray*}& & {R}^{2}= \frac{(\sum _{i=1}^{n}({\mathrm{obs}}_{i}-{\mu }_{\mathrm{obs}})({\mathrm{pred}}_{i}-{\mu }_{\mathrm{pred}}))^{2}}{\sum _{i=1}^{n}({\mathrm{obs}}_{i}-{\mu }_{\mathrm{obs}})^{2}\sum _{i=1}^{n}({\mathrm{pred}}_{i}-{\mu }_{\mathrm{pred}})^{2}} \end{eqnarray*}

(2) \begin{eqnarray*}& & \mathrm{MAE}= \frac{1}{n} \sum _{i=1}^{n}{|}{\mathrm{obs}}_{i}-{\mathrm{pred}}_{i}{|}\end{eqnarray*}

(3) \begin{eqnarray*}& & \mathrm{RMSE}=\sqrt{ \frac{1}{n} }\sum _{i=1}^{n}({\mathrm{obs}}_{i}-{\mathrm{pred}}_{i})^{2}\end{eqnarray*}

where *n* represents the total number of sampled genomes, obs_i_ corresponds to the number of reads measured for a specific genome, pred_i_ is the predicted value of reads, *i* represents each individual genome, and µcorresponds to the mean. The method is shown in a flow chart in Fig. 1.

### Creation of the dashboard

Since the machine learning model was trained in Python, we created a dashboard developed in this language. The source code was packaged in an image using Docker (Anderson, 2015) containers. This image was uploaded to an open-source cloud platform as a web application for other researchers to use. This dashboard has three sliders and a textbox for “Concentration of cDNA (ng/µl)”, “Coverage depth (mean)” and “Coverage per genome (percentage)” that is updated when the user changes these objects. Also, input values are displayed in a figure with all values used for model training. Finally, users can verify the value of the prediction reads together with the local explanation graph; in this way, every time a value is modified in the slider, the prediction, and the graphs are automatically updated. The application also allows the saving of the graphs obtained with each prediction thanks to interactive buttons that are displayed on each graph. The web application can be accessed at: https://genomicdashboard.herokuapp.com/.

### Ethical considerations

The study was approved by the Ethics Committee of Universidad del Valle, Colombia, with code 188-020, samples and database were anonymized.

## Results

### Data analysis

**Figure 2:**
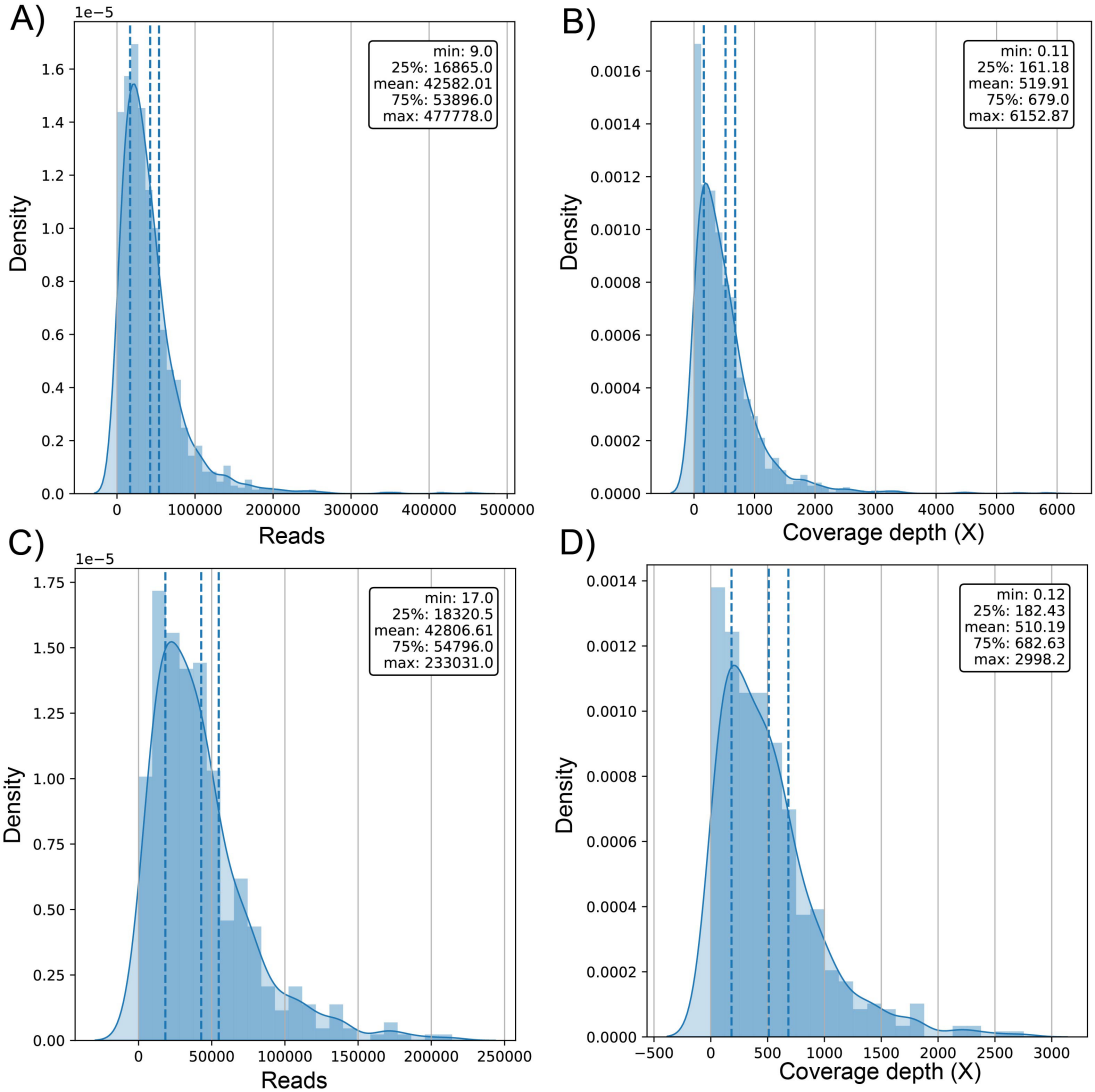
Histogram of distribution of sequenced reads and coverage depth in the dataset of 1461 (A and B, respectively) and 471 genomes (C and D, respectively).

The average number of reads in the evaluated genomes was 42.58k ± 42.9k for dataset (1) and 42.81k ± 34.93k for dataset (2) (Table S1) with a positively skewed distribution in both datasets (Figs. 2A and 2C). In dataset (1), outliers were on both sides of log e scale boxplots, mainly accumulated on the lower side, while dataset (2) has values exclusively in that space (Fig. S1). The average coverage depth per genome was 519.91 ± 563.5X and 510.2 ± 453.22X for dataset (1) and dataset (2), respectively (Table S1), with an almost identical distribution (Figs. 2B and 2D).

**Figure 3:**
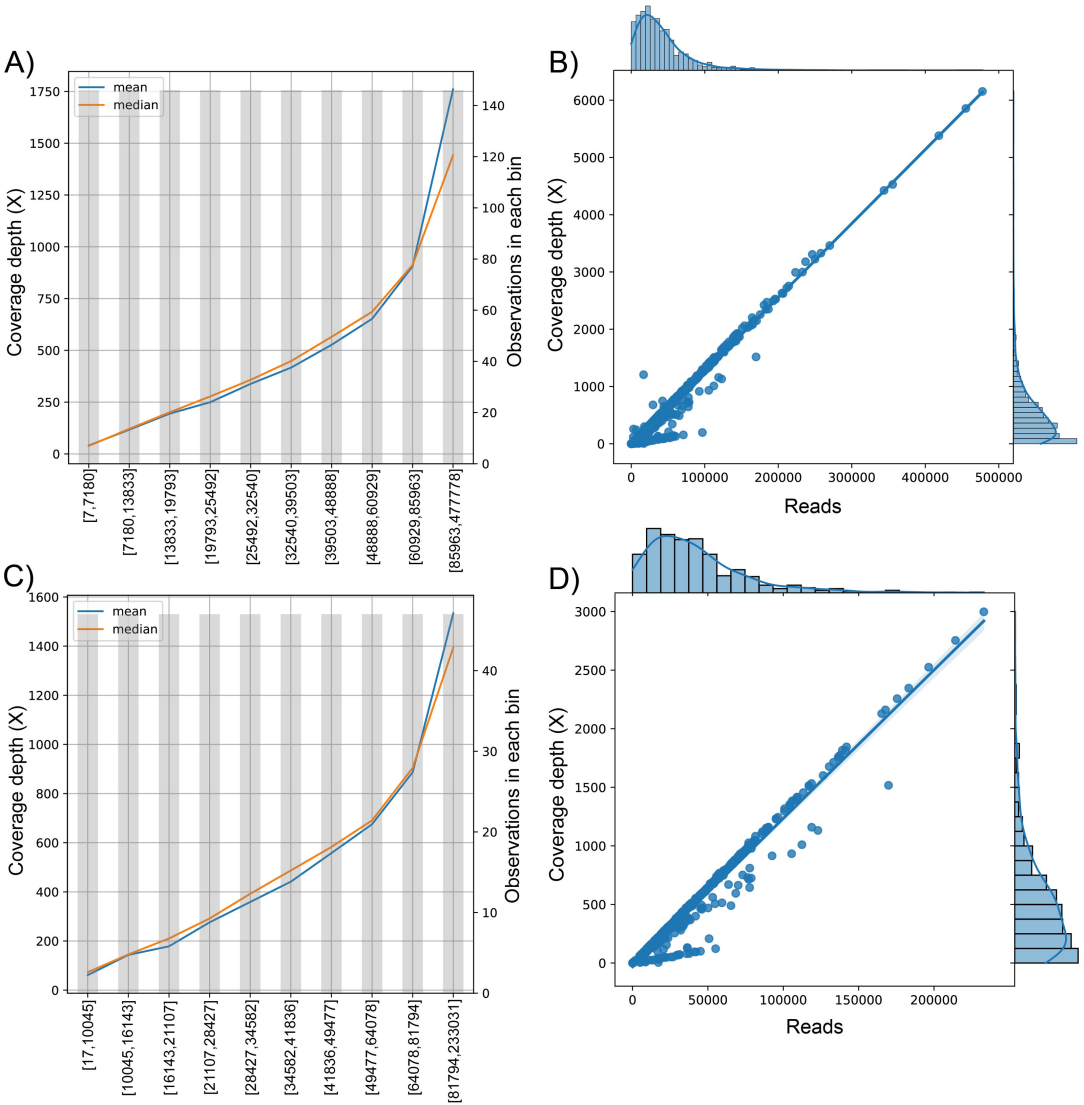
The behavior of the target variable (number of reads per genome) for coverage depth in the dataset 1 (A) and dataset 2 (C). Scatter plot with the distributions of the two variables in dataset 1 (B) and dataset 2 (D). The plot of sequenced reads into bins comparing the mean and median value of coverage depth data in each bin.

We analyzed the correlation between the target variable (number of reads sequenced) and the dependent variable (coverage depth) (Fig. 3). Since the curve was not flat, the feature was predictive and could be used for model construction (Figs. 3A and 3C). This trend is shown in the univariate regression of the number of reads compared to coverage depth, pointing to a strong direct relationship between these features (Figs. 3B and 3D) with a Pearson’s Correlation Coefficient of 0.977 and 0.959 for dataset (1) and dataset (2), respectively (*p*-value <0.001 in both cases).

We found the relationship in the dataset (1): *y* = 0.0129x − 30.315; for every 100 reads added in real-time in an experiment, the expected average coverage depth of each sample increases by 1.3X. Therefore, if we desire 200X depth for lineage assignment, we need at least 17,854 reads.

### Models’ performance

The performance metrics values for all models tested showed R^2^ between 0.93 and 0.98 (Table 1). The ensemble models had better performance in both datasets with an R^2^ of 0.9794 for GBR in dataset1 and 0.9506 for RFR in dataset2; moreover, SVR showed in both cases the lowest R^2^ values (0.9646 and 0.9347, respectively) (Table 1). In the case of dataset1, GBR was followed by models LssR, RFR, then SVR. However, the performance order of the models changed when we used the smaller dataset (2), with the best being RFR, followed by GBR, LssR, then SVR. This performance pattern is similar when the average R^2^ of cross-validation is evaluated, where GBR outperforms the rest of the models with an R^2^ of 0.9771 and only changes radically in dataset 2, where LssR is the highlight as the best fitting, with 0.9637, following by RFR, SVR, and GBR. RFR and SVR presented the least mean absolute error of predictions in both datasets (2560 and 3875 sequenced reads for dataset (1) and dataset (2), respectively: Table 1).

**Table 1:** R^2^, RMSE, and MAE performance values of dataset 1 and 2 evaluated under different models.

**Dataset**	**Model**	**Average R^2^**	**R^2^**	**MAE**	**RMSE**
1	LssR	0.9715	0.9741	3817.5526	6509.1245
	SVR	0.9610	0.9646	2762.9007	7607.8926
	GBR	**0.9771**	**0.9794**	2843.2006	**5804.2179**
	RFR	0.9756	0.9727	**2559.9966**	6683.7374
2	LssR	**0.9637**	0.9411	5191.3255	9082.4665
	SVR	0.9539	0.9347	**3874.7963**	9565.7193
	GBR	0.9539	0.9497	5099.3944	8393.9312
	RFR	0.9603	**0.9506**	4478.2003	**8317.3773**

**Notes.**

The best values for each metric are in bold.

We used GBR for the construction of the predictive app of variables modulating the total number of sequenced reads. In short, the best performance models (GBR and RFR) explain 95–98% of the variance of total reads of genomes in both used datasets. On average, predictions for these models in dataset (1) and dataset (2) have a mean error of 2698 (12%) and 4779 (18%) sequenced reads, respectively.

**Figure 4:**
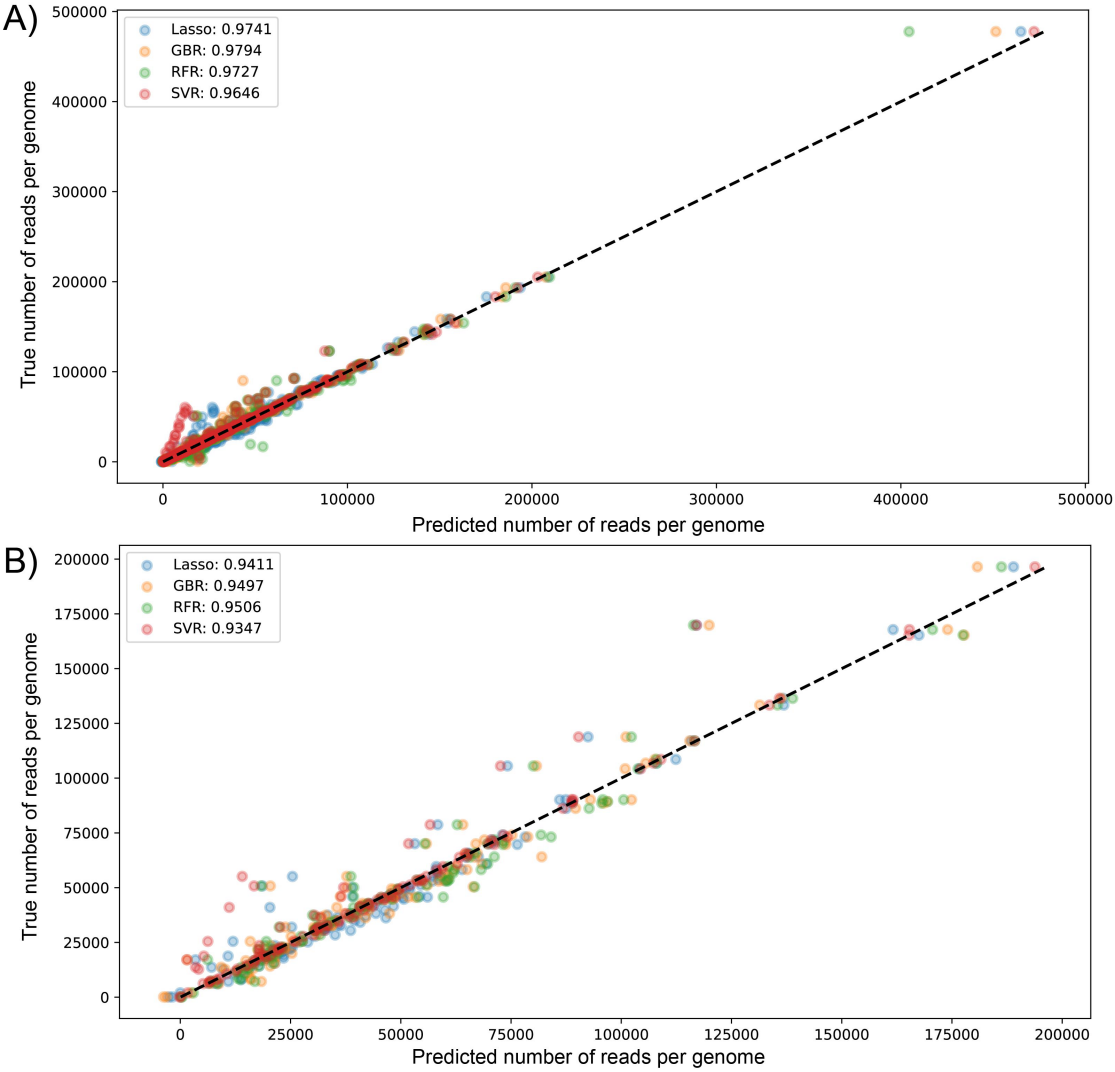
Scatter plot for predicted *vs* measured (true) values for sequenced reads, using linear regression with Lasso regularization, Support Vector Regressor, Gradient Boosting, and Random Forest for two datasets of 1461 (A) and 471 genomes (B). R^2^ scores are equivalent to Table 1 values, obtained from test partitions of each dataset. “Average R^2^” values obtained from the geometric mean of the scores of 5-iterations of cross-validation.

We visualized the results of k-fold validation by plotting predicted values against the actual sequenced reads, observing that points are close to a diagonal line where predicted = real (Figs. S2 and S3). Consistent with the performance metrics, the ensemble models presented better predictive power and fitting compared to other algorithms. Figure 4 shows the high similarity in predicted reads values between models in both datasets, with SVR having more outliers compared to the rest, underestimating the true values of the target variable.

The biggest error in the test set was over 73,353 and 53,444 sequenced reads modeled by RFR for dataset (1) and dataset (2), respectively. Figs. S4 and S5 visualize the errors in both datasets by plotting predicted against the residual of each prediction; for dataset (1), most errors lie on the negative side, meaning these predictions are underestimated (composed of 61.20, 51.91, 57.92, and 60.38% of residuals in LssR, SVR, GBR, and RFR, respectively). Similarly, dataset (2) has 70.34, 53.40, 61.01, and 70.34% negative residuals, respectively. In both datasets, SVR showed the most equitable distribution of positive and negative errors. Moreover, LssR and SVR showed a slight positive skew with many values in the interval of 10,000 to 30,000 compared to ensemble models that have more randomly distributed errors, which is consistent with a more approximately normal distribution of residuals in GBR and RFR compared to LssR and SVR (Figs. S6 and S7).

Finally, the plausibility of the GBR model predictions for both datasets was evaluated. In the case of the dataset (1), the model predicts 43,089 reads were obtained (42,740 true reads), given cDNA quantification values between 14.85–24.20 ng/uL, coverage between 91.12–96.03, and coverage depth between 380-688X. In dataset (2), the model predicts that 78,807 reads were obtained (73,161 true reads), given N2 CT between 13-16, cDNA quantification values above 34.8 ng/uL, coverage between 91.18–95.28, and coverage depth above 643X.

## Discussion

The objective of this study was to test multiple SML regression algorithms to accurately estimate enough sequenced reads per SARS-CoV-2 genome to achieve an acceptable depth of coverage given different sample variables. Therefore, we compared simple linear regression with parametric models SVR and ensemble algorithms for the accuracy and robustness of the predicted target variable. The sample variables of the mean number of sequenced reads, mean coverage per genome and CT have literature-supported relationships (Liu et al., 2022; Wang et al., 2011). Both genome coverage and depth of coverage are highly dependent on the viral concentration of the sample, Gauthier et al. (2021) showed that RT-qPCR SARS-CoV-2 samples with CTs greater than 25 tend to have coverage less than 95%. Similarly, Brinkmann et al. (2021) reported that using 75,000 reads with a depth >10X, they achieved > 98% coverage of the SARS-CoV-2 genome using ONT. Consequently, including these variables was a priority to develop and create a machine learning model, from data gathering to evaluation.

Estimated performance metrics show GBR had the highest R^2^ and lowest RMSE values for predicted reads in the test and training datasets, indicating this ensemble model can provide accurate depth estimations. However, this performance order changes in average R^2^ of cross-validation in dataset 2, indicating that GBR (where ${\mathrm{R}}_{\mathrm{test}}^{2}\lt {\mathrm{R}}_{\mathrm{training}}^{2}$) does not generalize well or has poor stability in this dataset (Li, Luan & Wu, 2020); however, this limitation can be attributed to small sample size. On the other hand, MAE in both tested datasets was reduced in RFR and SVR models, outperforming GBR (Table 1). This pattern can be attributed to the nature of RMSE, which penalizes large gaps in the model predictions, while MAE does not (Koutsandreas et al., 2021). On average, RFR and SVR have more large-scale errors but fewer small-scale errors than GBR; this can be visualized in the residuals plots (Figs. S4 and S5), where GBR accumulates more small-scale errors in the interval of 10,000 to 30,000 reads, while SVR and RFR have more large-scale errors randomly distributed.

Most of the sequencing input variables used in this study are correlated. Due to this, GBR was preferred over other models that are more sensitive to collinearity, such as SVR (Cutler et al., 2007). Moreover, both the adaptability of ensemble models to small sample sizes along with the insensitivity to overfitting data, outliers, and less predictive input (when the depth of coverage function was removed from data set 2) were advantages (Bellido-Jiménez, Gualda & García-Marín, 2021; Wang et al., 2016).

Finally, we propose the use of Gradient Boosting Regressor for real-time monitoring of ONT MinION sequencing of SARS-CoV-2 samples. The prediction accuracy of the method should be further validated by optimizing the modeling algorithms. Therefore, the online dashboard was created using GBR to predict the mean depth of coverage and other variables from simple input (number of sequenced reads), to improve the cost-effectiveness of flow cells and ONT sequencing for laboratories with fewer resources. It would be interesting to apply the method to monitor other reactions *a priori* sequencing parameter as storage of sample time, quality metrics of cDNA, and different primer kits to verify reproducibility. This research contributes to the establishment of SARS-CoV-2 genomic surveillance management strategies for simple experiment monitoring and precise modeling methods.

## Conclusions

We propose a novel method for the prediction of reads necessary to sequence SARS-CoV-2 at a sufficient coverage depth that allows database-acceptable lineage. Different machine learning models were trained; the Gradient Boosting Regression algorithm showed the best fit, explaining more than 98% of the variance of the sequenced reads, and presented an average error of 16% in the predictions. The Gradient Boosting Regressor provides a useful exploratory and predictive tool for estimating reads given *a priori* sequencing process variables such as CT (gene N) and amount of cDNA per sample and *a posteriori* sequencing quality variables coverage and mean depth of coverage per genome. The implementation of these methods will bring a reduction in sequencing costs through process optimization.

##  Supplemental Information

10.7717/peerj.14425/supp-1 Table S1 Descriptive statistics of five variables obtained from SARS-CoV2 genome sequencing with good lineage assignment for two datasets (dataset 1, 1461 samples; dataset 2, 471 samples)

10.7717/peerj.14425/supp-2 Figure S1 Box and whisker plot using natural logarithmic scale of sequenced reads and coverage depth in the dataset 1 (A and B, respectively) and dataset 2 (C and D, respectively) The *x*-axis depicts sequenced reads and coverage depth, while the *y*-axis depicts values in natural logarithmic scale. Each dot represents outliers in the data set.

10.7717/peerj.14425/supp-3 Figure S2 Scatter plot for predicted vs measured values for sequenced reads, using linear regression with LssR (A), SVR (B), GBR (C), and RFR (D) for 1461 genomes (dataset 1), using five k-fold cross-validation

10.7717/peerj.14425/supp-4 Figure S3 Scatter plot of predicted vs measured values for sequenced reads, using linear regression with LssR (A), SVR (B), GBR (C), and RFR (D) for 471 genomes (dataset 2), using five k-fold cross-validation

10.7717/peerj.14425/supp-5 Figure S4 Plot of predicted vs residuals for sequenced reads, using linear LssR (A), SVR (B), GBR g (C), and RFR (D) for 1461 genomes (dataset 1) Maximum errors were 34127, 48415, 46741, and 73353 residuals values of reads for LssR, SVR, GBR and RFR, respectively.

10.7717/peerj.14425/supp-6 Figure S5 Plot of predicted vs residuals for sequenced reads, using linear LssR (A), SVR (B), GBR g (C), and RFR (D) for 471 genomes (dataset 2) Maximum errors were 52798, 52752, 49899, and 53444 residuals values of reads for LssR, SVR, GBR and RFR, respectively.

10.7717/peerj.14425/supp-7 Figure S6 Density distribution of residual of sequenced reads values, using LssR (A), SVR (B), GBR (C), and RFR (D) for 1461 genomes (dataset 1)

10.7717/peerj.14425/supp-8 Figure S7 Density distribution of residual of sequenced reads values, using LssR (A), SVR (B), GBR (C), and RFR (D) for 471 genomes (dataset 2)

## Additional Information and Declarations

### Data Availability

The following information was supplied regarding data availability: The code is available at GitHub: https://github.com/TAOLabUV/PredictION. The dataset is available at Zenodo: David E. Valencia-Valencia, Diana Carolina Lopez Alvarez, Nelson Rivera-Franco, Andres Castillo, Johan S Piña, Carlos A Pardo, & Beatriz Parra. (2022). PredictION: A predictive model to establish the performance of Oxford sequencing reads of SARS-CoV-2 [Data set]. Zenodo. https://doi.org/10.5281/zenodo.7104956.
