# Supplementary material for: PredictION: a predictive model to establish the performance of Oxford sequencing reads of SARS-CoV-2
Source: PeerJ. 2022 Nov 30;10:e14425. doi: 10.7717/peerj.14425 (PMC9744141; doi:10.7717/peerj.14425)
Supplement: Table S1 [file peerj-10-14425-s001.docx]

|  |  | **Concentration of cDNA**  **(ng/µl)** | **Coverage depth**  **(mean)** | **Number of Sequenced Reads** | **Coverage per genome (Percentage)** | **CT**  **(N2 Gene)** |
| --- | --- | --- | --- | --- | --- | --- |
| **d1** | Mean | 29.64 | 519.91 | 42582.01 | 89.38 |  |
|  | std | 20.97 | 563.50 | 42879.65 | 19.53 |  |
|  | Min | 1.00 | 0.11 | 9.00 | 0.00 |  |
|  | 0.25 | 15.00 | 161.18 | 16865.00 | 91.19 |  |
|  | Median | 24.20 | 380.15 | 32540.00 | 96.02 |  |
|  | 0.75 | 39.00 | 679.00 | 53896.00 | 98.68 |  |
|  | Max | 120.00 | 6152.87 | 477778.00 | 100.00 |  |
| **d2** | Mean | 29.94 | 510.19 | 42806.61 | 90.94 | 16.13 |
|  | std | 21.03 | 453.22 | 34931.28 | 16.34 | 4.95 |
|  | Min | 2.28 | 0.12 | 17.00 | 0.00 | 0.00 |
|  | 0.25 | 15.80 | 182.43 | 18320.50 | 91.35 | 13.00 |
|  | Median | 23.80 | 412.30 | 34582.00 | 95.87 | 15.70 |
|  | 0.75 | 36.40 | 682.63 | 54796.00 | 98.68 | 19.05 |
|  | Max | 120.00 | 2998.20 | 233031.00 | 100.00 | 33.20 |
