# Supplementary figures and images for: PredictION: a predictive model to establish the performance of Oxford sequencing reads of SARS-CoV-2
Source: PeerJ. 2022 Nov 30;10:e14425. doi: 10.7717/peerj.14425 (PMC9744141; doi:10.7717/peerj.14425)

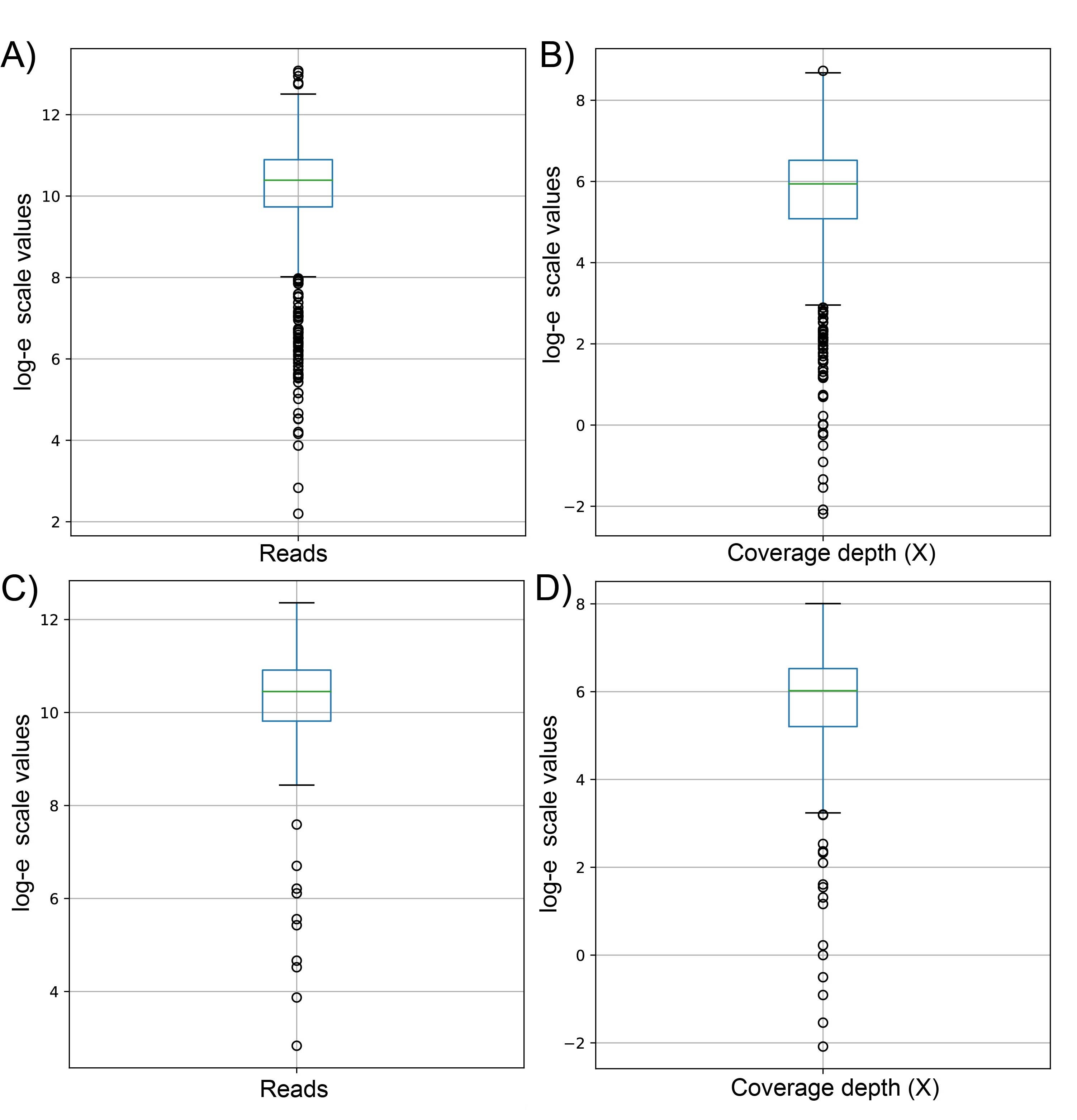

Supplement: Figure S1 — The x-axis depicts sequenced reads and coverage depth, while the y-axis depicts values in natural logarithmic scale. Each dot represents outliers in the data set. [file peerj-10-14425-s002.jpg]

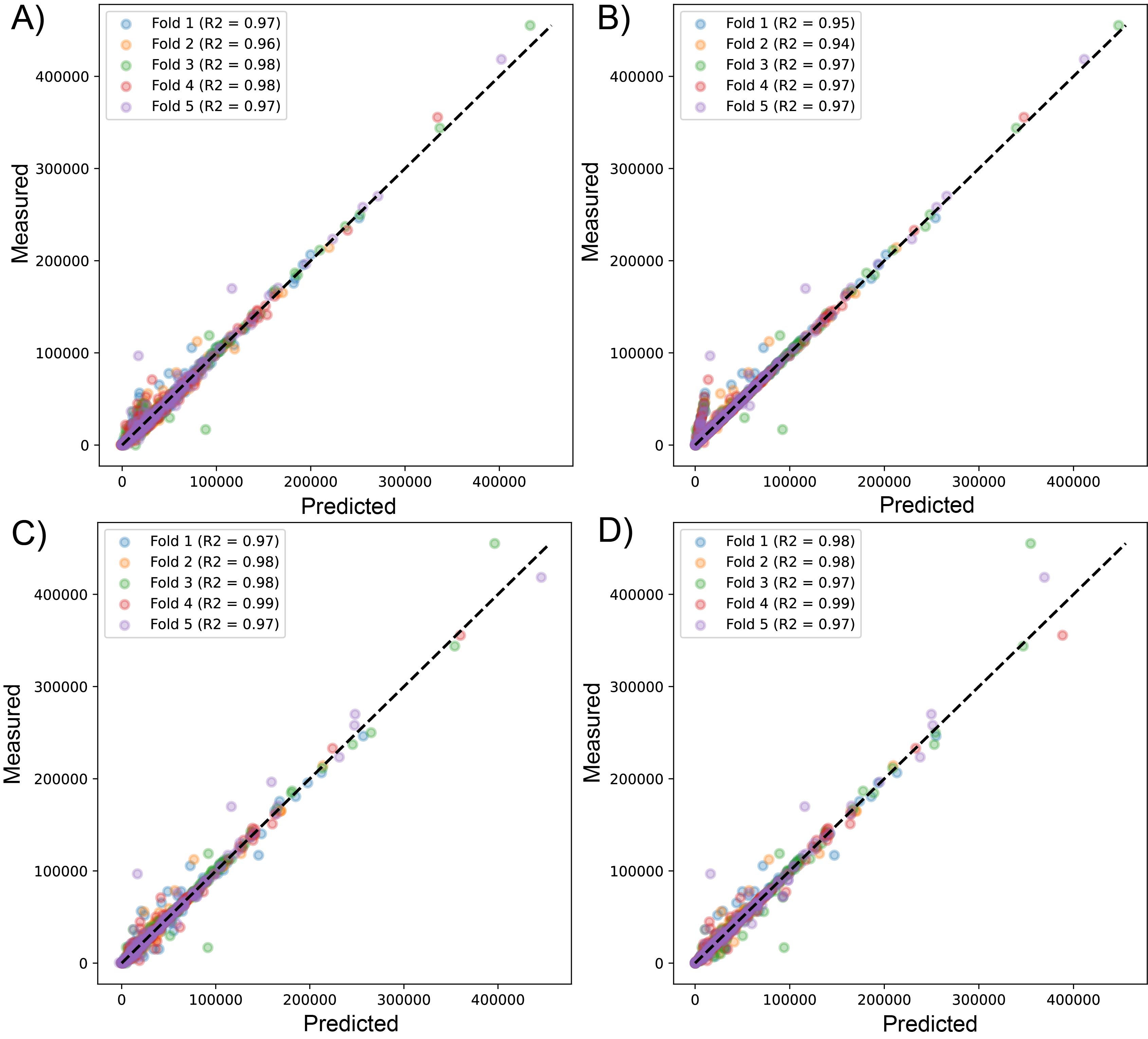

Supplement: Figure S2 [file peerj-10-14425-s003.jpg]

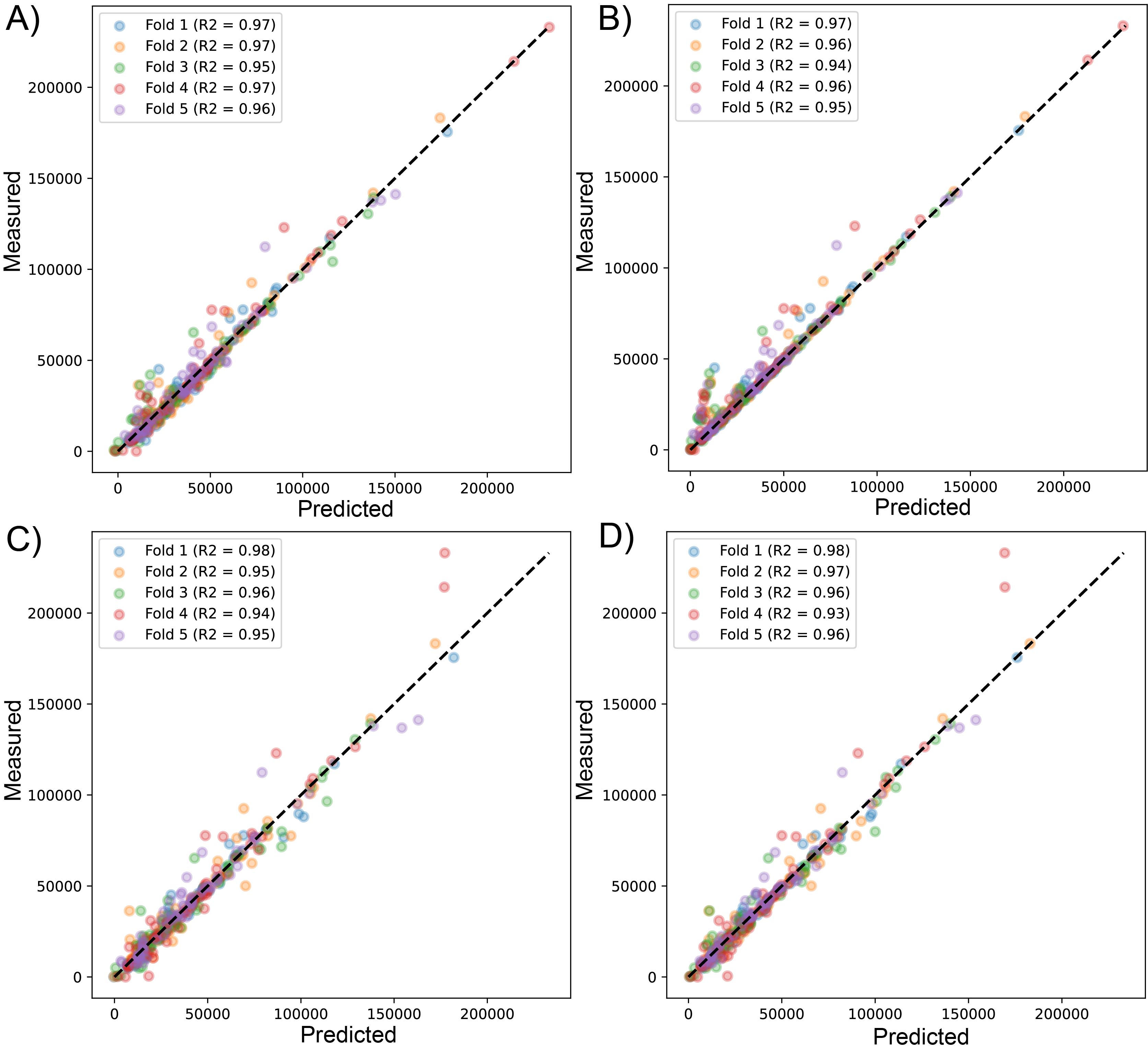

Supplement: Figure S3 [file peerj-10-14425-s004.jpg]

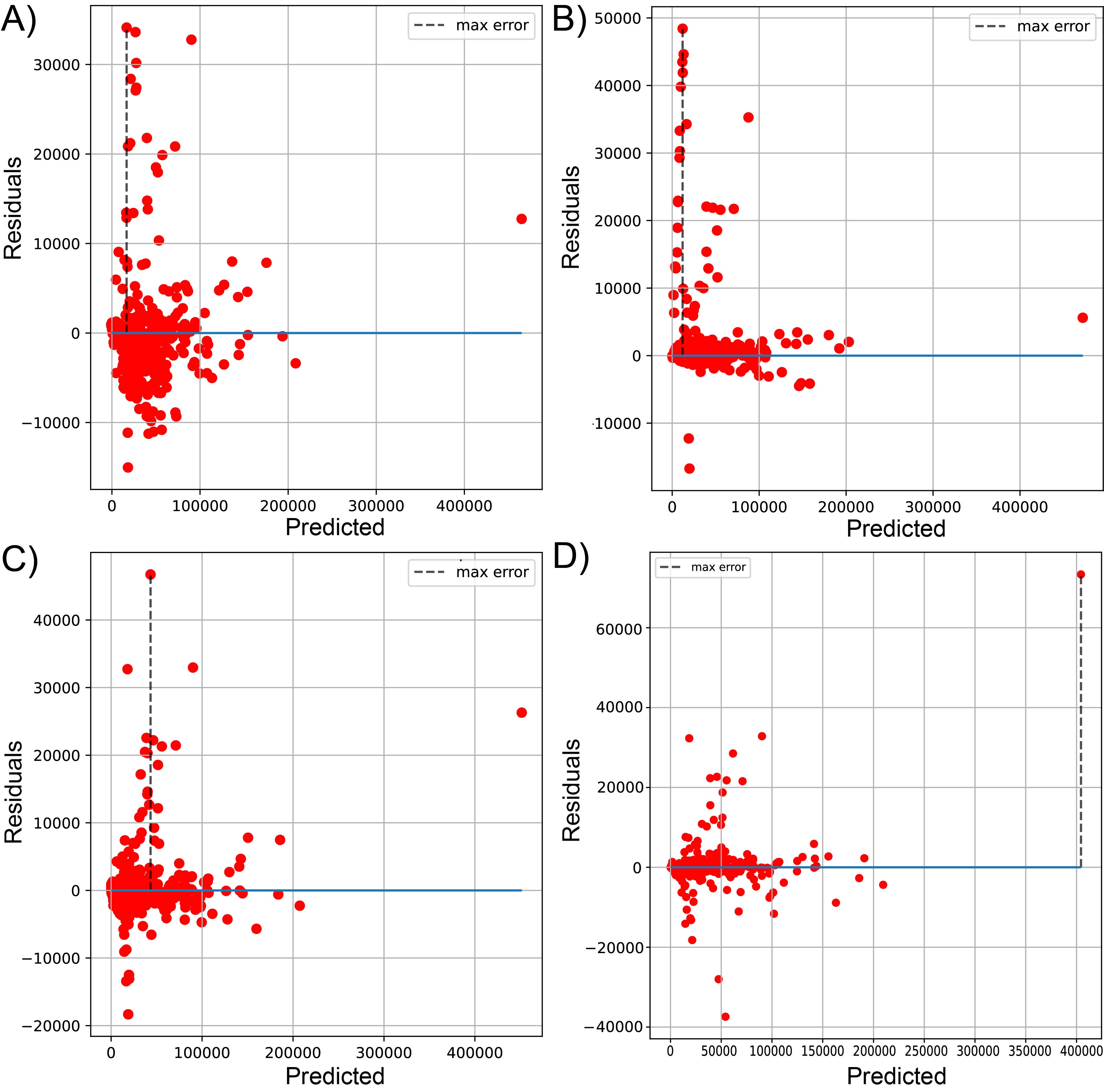

Supplement: Figure S4 — Maximum errors were 34127, 48415, 46741, and 73353 residuals values of reads for LssR, SVR, GBR and RFR, respectively. [file peerj-10-14425-s005.jpg]

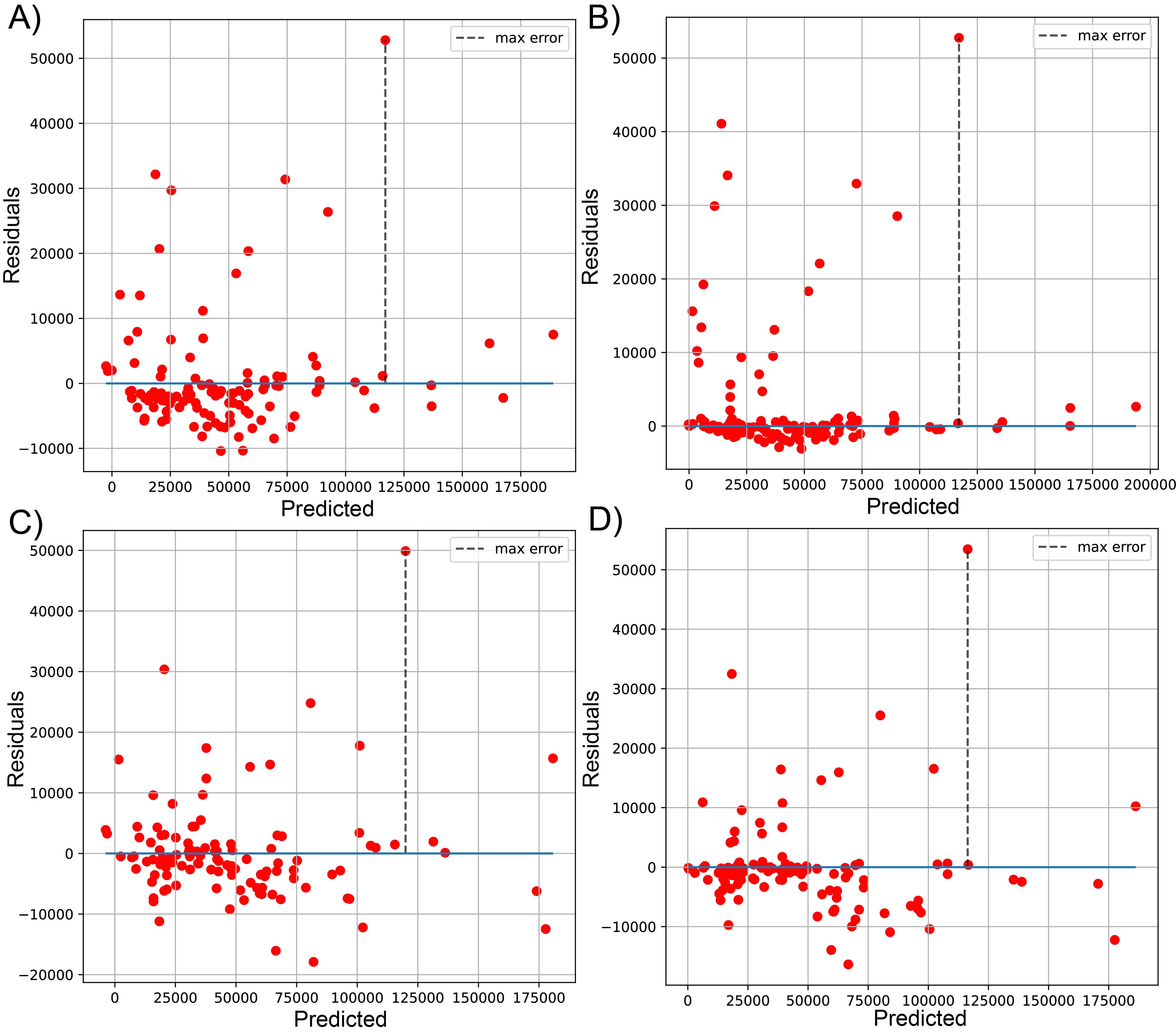

Supplement: Figure S5 — Maximum errors were 52798, 52752, 49899, and 53444 residuals values of reads for LssR, SVR, GBR and RFR, respectively. [file peerj-10-14425-s006.jpg]

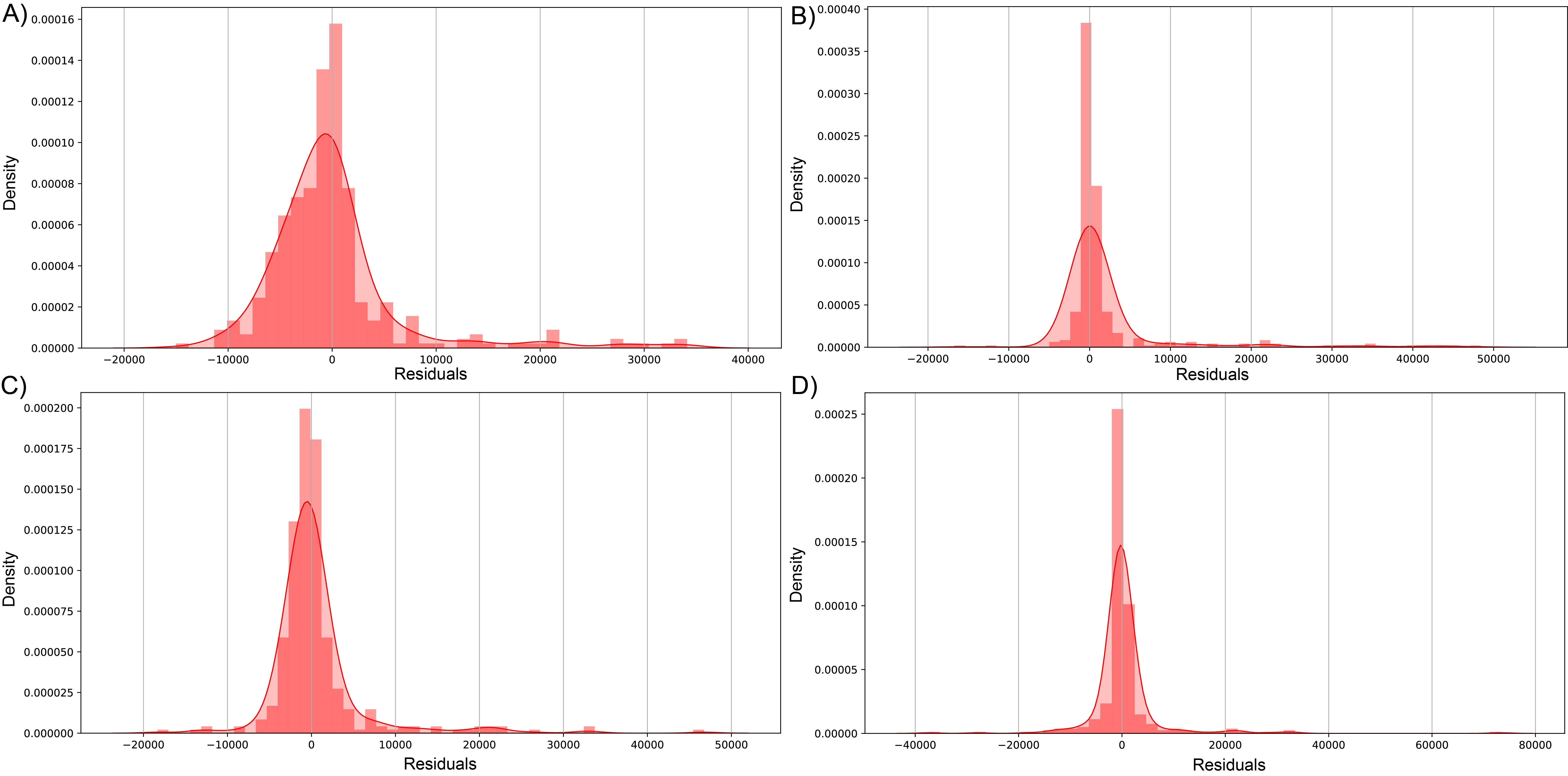

Supplement: Figure S6 [file peerj-10-14425-s007.jpg]

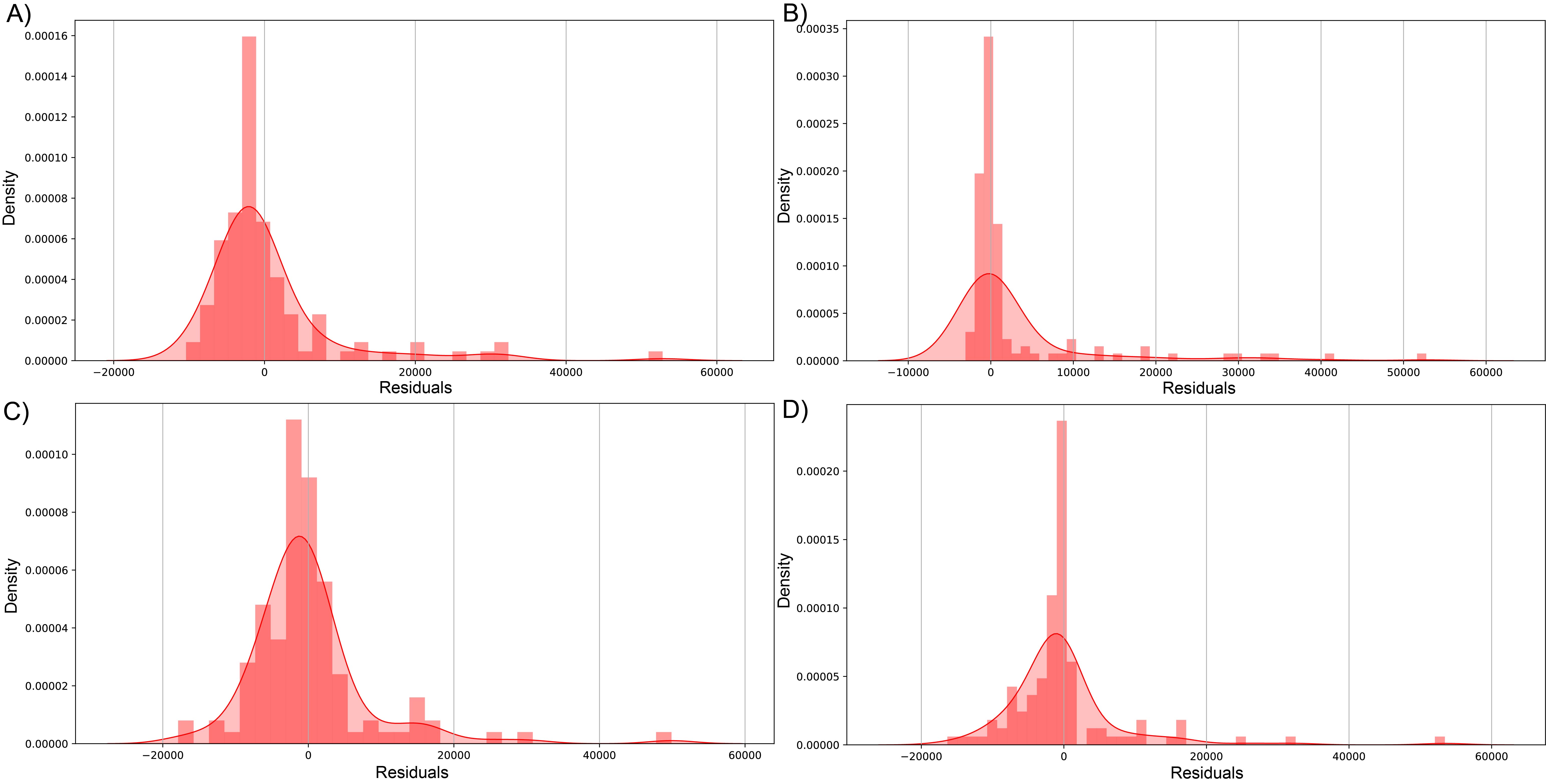

Supplement: Figure S7 [file peerj-10-14425-s008.jpg]
